# Cardioprotective Regimen of Adaptation to Chronic Hypoxia Diversely Alters Myocardial Gene Expression in SHR and SHR-mt^BN^ Conplastic Rat Strains

**DOI:** 10.3389/fendo.2018.00809

**Published:** 2019-01-22

**Authors:** Iveta Nedvedova, David Kolar, Jan Neckar, Martin Kalous, Michal Pravenec, Jan Šilhavý, Vlasta Korenkova, Frantisek Kolar, Jitka M. Zurmanova

**Affiliations:** ^1^Department of Physiology, Faculty of Science, Charles University, Prague, Czechia; ^2^Institute of Physiology, Czech Academy of Sciences, Prague, Czechia; ^3^Department of Cell Biology, Faculty of Science, Charles University, Prague, Czechia; ^4^Institute of Biotechnology, Czech Academy of Sciences, Prague, Czechia

**Keywords:** SHR, conplastic strain, SHR-mt^BN^, left ventricle, hypoxia, metabolism

## Abstract

Adaptation to continuous normobaric hypoxia (CNH) protects the heart against acute ischemia/reperfusion injury. Recently, we have demonstrated the infarct size-limiting effect of CNH also in hearts of spontaneously hypertensive rats (SHR) and in conplastic SHR-mt^BN^ strain characterized by the selective replacement of the mitochondrial genome of SHR with that of more ischemia-resistant Brown Norway rats. Importantly, cardioprotective effect of CNH was more pronounced in SHR-mt^BN^ than in SHR. Thus, here we aimed to identify candidate genes which may contribute to this difference between the strains. Rats were adapted to CNH (FiO_2_ 0.1) for 3 weeks or kept at room air as normoxic controls. Screening of 45 transcripts was performed in left ventricles using Biomark Chip. Significant differences between the groups were analyzed by univariate analysis (ANOVA) and the genes contributing to the differences between the strains unmasked by CNH were identified by multivariate analyses (PCA, SOM). ANOVA with Bonferroni correction revealed that transcripts differently affected by CNH in SHR and SHR-mt^BN^ belong predominantly to lipid metabolism and antioxidant defense. PCA divided four experimental groups into two main clusters corresponding to chronically hypoxic and normoxic groups, and differences between the strains were more pronounced after CNH. Subsequently, the following 14 candidate transcripts were selected by PCA, and confirmed by SOM analyses, that can contribute to the strain differences in cardioprotective phenotype afforded by CNH: Alkaline ceramidase 2 (*Acer2*), Fatty acid translocase (*Cd36)*, Aconitase 1 (*Aco1*), Peroxisome proliferator activated receptor gamma (*Pparg)*, Hemoxygenase 2 (*Hmox2)*, Phospholipase A2 group IIA (*Ppla2g2a*), Dynamin-related protein (*Drp*), Protein kinase C epsilon (*Pkce*), Hexokinase 2 (*Hk2)*, Sphingomyelin synthase 2 (*Sgms2)*, Caspase 3 (*Casp3*), Mitofussin 1 (*Mfn1*), Phospholipase A2 group V (*Pla2g5*), and Catalase (*Cat*). Our data suggest that the stronger cardioprotective phenotype of conplastic SHR-mt^BN^ strain afforded by CNH is associated with either preventing the drop or increasing the expression of transcripts related to energy metabolism, antioxidant response and mitochondrial dynamics.

## Introduction

Hypertension represents one of the major risk factors for the development of ischemic heart disease (1). The most extensively studied experimental model of essential hypertension is an inbred strain of spontaneously hypertensive rats (SHR). This strain develops hypertension during the 10–15th postnatal week (2). SHR are also frequently used for metabolic syndrome studies due to the development of insulin resistance, hyperinsulinemia, glucose intolerance, and hypertriglyceridemia (3, 4). Moreover, SHR manifested an increased sensitivity to ischemia/reperfusion (I/R) injury when compared to Wistar-Kyoto strain (WKY) (5, 6).

It has been repeatedly shown that cardiac resistance to I/R injury is tightly related to mitochondrial status and energy homeostasis of cardiomyocytes (7). In compliance with this knowledge a unique model of the SHR-mt^BN^ conplastic strain was developed by selective replacement of the mitochondrial genome of the SHR by the mitochondrial genome of normotensive, more ischemia resistant, Brown Norway rat strain (8). Recently, we have shown that the infarct size-limiting effect afforded by adaptation to continuous normobaric hypoxia (CNH; inspired oxygen fraction 0.1) was more pronounced in SHR-mt^BN^ than in progenitor SHR and correlated with the decreased sensitivity to mitochondrial permeability transition pore (mPTP) opening in both strains (9). Indeed, affection of mitochondria determines the cell fate during early phases of reperfusion by mPTP opening (10) as the result of reactive oxygen species (ROS) overproduction and calcium overload [reviewed in (11)]. Likewise, we have showed that mitochondrial antioxidants play an important role in cardioprotective phenotype induced by CNH in normotensive rats (12). Beside that both glucose and lipid metabolism signaling pathways have been shown to contribute to cardioprotective phenotype of chronically hypoxic hearts (13, 14).

Based on the above-mentioned data, we aimed first, to detect genes with significantly different mRNA expressions in progenitor SHR and conplastic SHR-mt^BN^ under normoxic and chronically hypoxic conditions using univariant analyses; and second, to identify candidate genes responsible for the differences between SHR and SHR-mt^BN^ after adaptation to CNH using multivariant analyses. These potential candidates may elucidate the relationship between CNH-afforded protection and mitochondrial genome.

## Materials and Methods

### Animals

Inbred progenitor SHR and its conplastic strain SHR-mt^BN^ (SHR harboring mitochondrial genome from Brown Norway rats) were used in present study. Both strains were exposed to CNH (inspired oxygen fraction 0.1) for 3 weeks and control groups were kept at normoxic conditions. The rats were housed at a 12/12-h light/dark cycle and fed by standard diet *ad libitum* and free access to the water. The animals were handled in accordance with the Guide for the Care and Use of Laboratory Animals published by the US National Institutes of Health (NIH Publication, 8th edition, revised 2011). The experimental protocol was approved by the Animal Care and Use Committee of the Institute of Physiology of the Czech Academy of Sciences.

### Tissue Preparation

All rats were killed by cervical dislocation in their environment, i.e., normoxic groups in room air and hypoxic groups in hypoxic chamber. The hearts were immediately excised and washed in ice-cold saline. Samples of left ventricle were rapidly frozen in liquid nitrogen and stored in −80°C until use.

### RNA Isolation and Chip Analyses

Total RNA isolation and reverse transcription was performed as described previously (12), with a slight modification. Briefly, RNA was isolated using RNAzol reagent (Sigma Aldrich) according to manufacturer's instructions. The purity of isolated RNA was tested on Agilent 2100. One microgram of total RNA was loaded to the reverse transcription and the PCR reaction was performed as described previously using RevertAid^TM^ H Minus First Strand cDNA Synthesis Kit with oligo(dT) primers (Fermentas). Gene-specific primers were designed using the Universal Probe Library Assay Design Center. The specific forward and reverse primer sequences are summarized in Supplement Table 1. At first, the samples for gene expression profiling were pre-amplified with 48 primers in 18 cycles with the following temperature profile: activation polymerase (95°C/3 min); amplification, 18 cycles of denaturation (95°C/15 s), and annealing (59°C/4 min) using iQ Supermix (Bio-Rad) and 2 μl cDNA (diluted on 10 ng input RNA). Subsequently, Biomark analysis were performed with following temperature profile: polymerase activation (95°C/3 min); amplification 30 cycles of denaturation (96°C/5 min), and annealing (60°C/20 s). Priming and pipetting were performed according to the manufacturer's instructions.

### Statistical Analysis

The quality of the quantification cycles (Cq) data of 48 mRNA transcripts from 4 experimental groups (SHR and SHR-mt^BN^ under normoxic and hypoxic conditions; *n* = 5) obtained from high-throughput qPCR instrument Biomark HD (Fluidigm) was checked by Fluidigm Real-Time PCR Analysis software (Fluidigm). The Cq data were basically processed by two approaches. First, the univariant analyses, based on the *p*-values, were used to reveal significant differences (*p* < 0.05) between four experimental groups within each mRNA transcript by ANOVA followed by Tukey's Multiple Comparison Posttest with Bonferroni correction using GenEx Enterprise (MultiD, SE) and GraphPad Prism software. Second, the multivariate principal component analysis analyses (PCA), based on the *p*-value and fold change of the gene, used auto scaled Cq data to reveal candidate genes responsible for diverse effects of CNH in SHR and SHR-mt^BN^. PCA is a powerful tool for reducing the dimensionality of a large data set in an unbiased way to identify clustering behavior. Subsequently, the Self-organizing maps (SOM) analysis was used to proof clustering obtained by PCA.

## Results

The 48 mRNA transcripts were analyzed as representatives of six groups of interest as follows: lipid metabolism, glucose metabolism, apoptosis, mitochondria, antioxidant defense and cell signaling (see Supplement Table 1). NormFinder analysis selected ribosomal protein *S18* with SD equal to 0.056, as the best reference gene from three candidates including hypoxanthine phosphoribosyltransferase 1 (*Hprt; SD* = 0.23) and beta-2-mikroglobulin (*B2m; SD* = 0.28).

### Univariate Analysis

**Figure 1:**
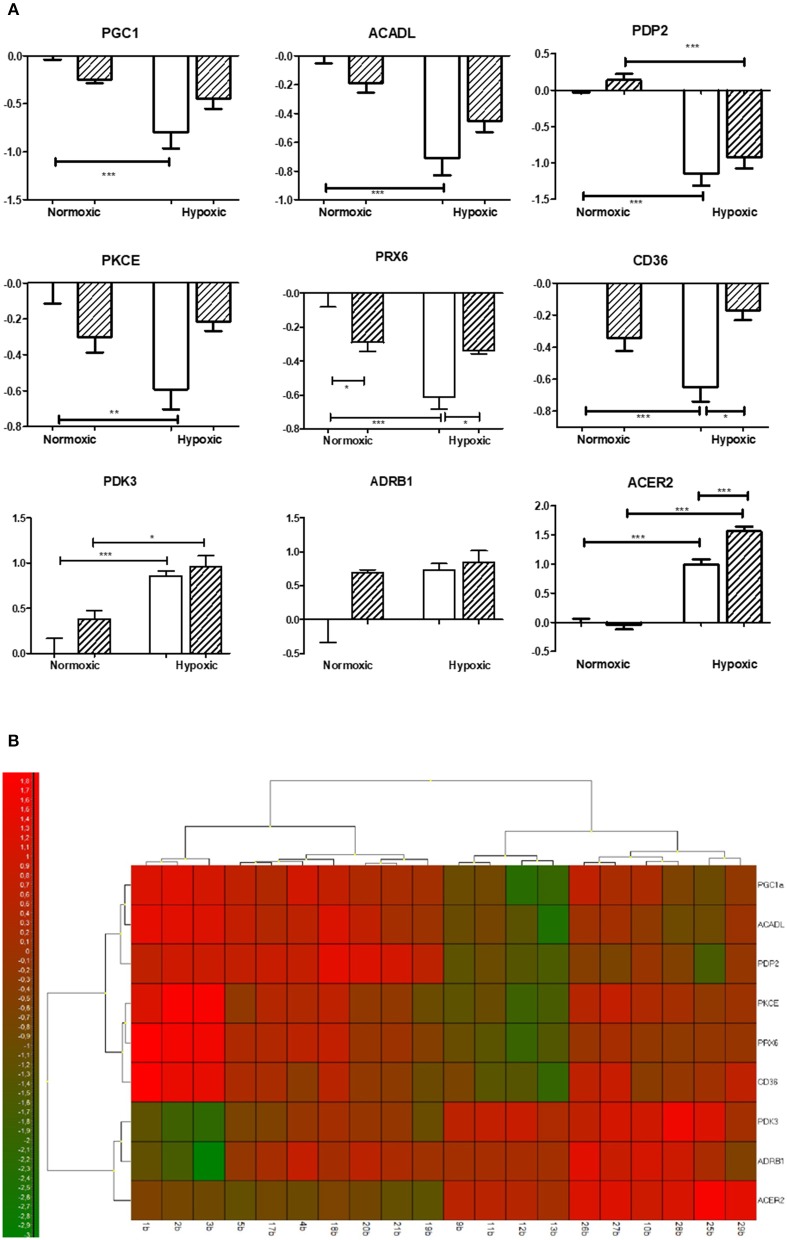
Effect of chronic continuous normobaric hypoxia on mRNA relative amount in the left ventricles of spontaneously hypertensive rats (SHR, empty bars) and its conplastic strain receiving mitochondria from normotensive Brown Norway rats (SHR-mtBN, hatched bars). Graphs showing genes with significant differences revealed by univariate analyses (ANOVA with Bonferroni correction) from 48 analyzed transcripts by Biomark Chip **(A)** and Heat map of all transcripts analyzed **(B)**. Values are mean ± SEM, (*n* = 5), *p*-value ^*^ ≤ 0.05, ^**^ ≤ 0.01, ^***^ ≤ 0.001.

The univariant analyses (with Bonferroni correction) revealed significant differences predominantly in lipid metabolism and mRNA transcripts related to oxidative stress (see Figure 1). The mRNAs related to glucose metabolism remained mostly unchanged, except for pyruvate dehydrogenase kinase 3 (*Pdk3)* and pyruvate dehydrogenase phosphatase (*Pdp2)*. Adaptation to CNH increased expression of *Pdk* and decreased expression of *Pdp2* compared to normoxic groups similarly in both SHR and SHR-mt^BN^ strains.

Regarding lipid metabolism, we observed a significant decrease in the expression of rate limiting enzyme of β-oxidation acyl-CoA dehydrogenase (*Acadl)* with a concomitant decline of fatty acid transporter (*Cd36)* in SHR but not in SHR-mt^BN^ after CNH. In contrast, CNH increased the expression of secretory phospholipases *Pla2g2a* and *Pla2g5a* in conplastic SHR-mt^BN^ compared to its normoxic counterpart. Interestingly, unlike *Pla2g2a, Pla2g4a* transcript level was lower in SHR-mt^BN^ group than in SHR under normoxic conditions. Moreover, alkaline ceramidase 2 (*Acer2)* expression increased after CNH in both strains, but the effect was more pronounced in SHR-mt^BN^ than in SHR.

Among the genes related to oxidative stress, CNH increased the expression of monoaminooxidase A (*MaoA)* and hemoxygenase 1 (*Hmox1)* in both strains. However, *Hmox1* was lower in SHR-mt^BN^ than in SHR under normoxia. The level of mRNA transcript of peroxiredoxin 6 (*Prx6)* was also lower in normoxic SHR-mt^BN^ than in SHR. CNH led to a significant drop of *Prx6* in SHR but not in SHR-mt^BN^, resulting in higher level in the later group.

Whereas we did not observe any changes in the level of transcripts related to apoptosis and mitochondria, the expression of peroxisome proliferator-activated receptor gamma coactivator 1 alpha (*Pgc1*) markedly decreased in chronically hypoxic SHR but not in corresponding SHR-mt^BN^. Protein kinase C epsilon (*Pkce*) transcript level was lower in normoxic SHR than in SHR-mt^BN^ and protein kinase C delta (*Pkcd)* significantly increased after CNH in SHR-mt^BN^ only.

### Multivariate Analysis

**Figure 2:**
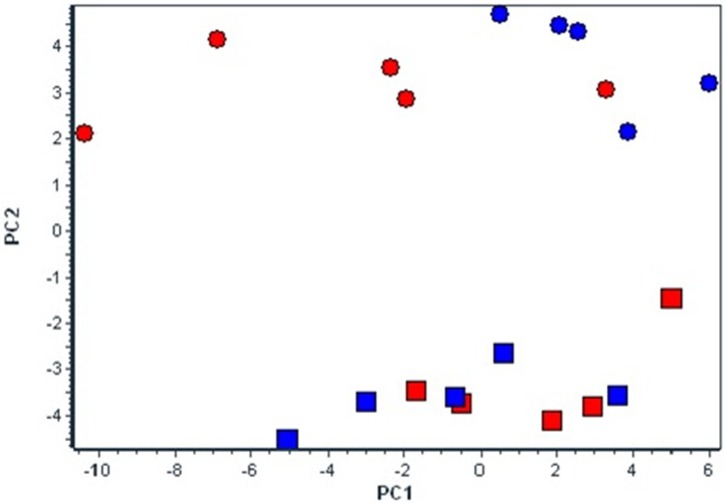
Effect of chronic continuous normobaric hypoxia on mRNA relative amount in spontaneously hypertensive rats (SHR, red) and its conplastic strain receiving mitochondria from normotensive Brown Norway rats (SHR-mtBN, blue) assessed by the multivariant principal component analysis (PCA) of all 48 genes. The diagram demonstrates clusters of selected candidate genes by PCA responsible for diverse effects of CNH in SHR and SHR-mt^BN^ under normoxic (rectangle) and CNH conditions (circle).

The basic PCA analysis of the whole set of 48 mRNA transcripts divided four experimental groups into two different clusters according to the experimental conditions, i.e., normoxia vs. hypoxia (Figure 2). Moreover, under hypoxic conditions the differences between strains became more pronounced.

**Figure 3:**
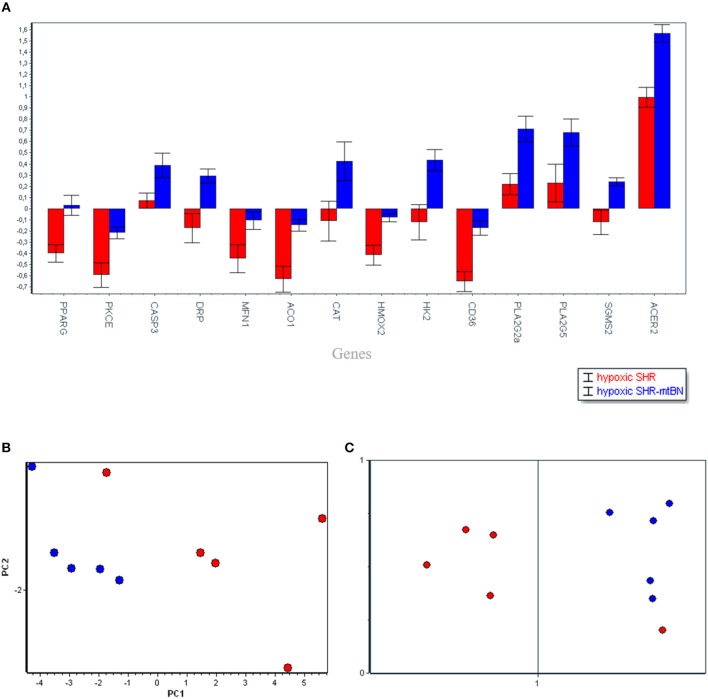
Selected candidate genes (14), responsible for the differences between spontaneously hypertensive rats (SHR, red) and its conplastic strain receiving mitochondria from normotensive Brown Norway rats (SHR-mtBN, blue) unmasked by adaptation to chronic continuous normobaric hypoxia (CNH) **(A)**. The distribution of selected genes assessed by principal component analysis (PCA) based on the *p*-value and fold change of the genes **(B)**; and confirmed by Self-organizing maps (SOM) **(C)**. Normoxic conditions (rectangle) and CNH conditions (circle).

Subsequent PCA (Figure 3) aimed to analyse the diverse effects of hypoxia on SHR and SHR-mt^BN^ strains, and selected most important candidate genes which might contribute to the strain differences in cardiopotective phenotype afforded by CNH according to increasing *p*-value as follows: *Acer2* (0.0013), *Cd36* (0.0021), *Aco1* (0.0057), *Pparg* (0.0061*), Hmox2* (0.0093), *Ppla2g2a* (0.0097), *Drp* (0.0115), *Pkce* (0.0145), *Hk2* (0.0155), *Sgms2* (0.0166), *Casp3* (0.0398), *Mfn1* (0.0491), *Pla2g5* (0.0611), *Cat* (0.0633). As seen above the PCA also selected two transcripts in which the p-value exceeded 0.05 level *(Pla2g5a* and *Cat)* however the influence of fold change became more important for selection of that genes by PCA. Further SOM analysis confirmed the clustering by PCA, except one sample.

## Discussion

Recently we demostrated that CNH adaptation of SHR and conplastic SHR-mt^BN^ induced cardioprotective phenotype in both strains which was more pronounced in SHR-mt^BN^ (9). In the present study we aimed to find out candidate genes which might contribute to the improved cardioprotective phenotype of hypertensive SHR-mt^BN^ strain after CNH. The univariant analyses revealed predominant role of lipid metabolism and antioxidant system in the effect of hypoxia. Beside that, attenuation of pyruvate transport into the mitochondria may suggest increasing contribution of anaerobic glycolysis in hypertensive heart of both strains. Interestingly, multivariant PCA and SOM analysis has identified 14 candidates out of 48 genes with predominant role of lipid metabolism.

The overview of the obtained data from both analyses (Figures 1, 3) shows that mitochondrial genome replacement led to two different alterations of SHR-mt^BN^ response to CNH: it either prevented drop of transcripts level induced by CNH or significantly increased upregulation of certain transcripts by CNH. The former effect was related to fatty acid transport and β-oxidation (*Cd36, Acadl)*, antioxidants *(Prx6, Hmox2, Aco1)*, signaling *(PKCe, Pgc1, Pparg)* and mitochondrial genes *(Mfn1, Pdp2)*. However, the latter effect was observed in most transcripts related to lipid metabolism *(Ppla2g2a, Pla2g5, Sgms2 Acer2)* and transcripts which all might be functionally related to mitochondria *(Casp3, Drp, Cat* and *Hk2)*.

### Glucose and Lipid Metabolism

In the present study we observed CNH-induced expression of *Hk2* in SHR-mt^BN^ in line with our recent studies which showed increased activity of Akt signaling pathway including hexokinase (HK) and glucose transporters (GLUTs) in SHR and SHR-mt^BN^ as well as in normotensive rats (13–15). Chronic hypoxia-induced shift in substrate utilization from fatty acids to glucose has been already reported (16, 17). However, we describe a novel mechanism which include an essential peroxisomal role in β-oxidation support utilizing the enhanced glycolytic flux (unpublished data) that also may correspond with present data. Indeed, beside an increased glucose-utilizing capacity of CNH myocardium, we suggest that the capability of glucose oxidative metabolism is attenuated in both hypertensive strains due to upregulation of *Pdk3* and downregulation of *Pdp2*.

Mitochondrial ability to process long-chain fatty acids (LCFAs) might be reduced in SHR after CNH due to downregulation of *Acadl*, while in conplastic SHR-mt^BN^ this effect was suppressed. Similarly, *Cd36* level dropped after CNH suggesting that FA influx mediated by *CD36* can be decreased in SHR. In contrast, mRNA level of both secretoric PLA2 (sPLA2; *Pla2g2a*, gene and its paralog *Pla2g5a)* increased in SHR-mt^BN^ hearts adapted to CNH and this change can be assumed as cardioprotective. However, the role of sPLA2 in myocardial susceptibility to I/R injury is rather controversial. Although the *Pla2g2a* gene deletion has been shown to correlate with infarct size expansion (18), increased plasma concentration of sPLA2 is considered as a marker of I/R injury in human (19, 20). Moreover, inhibition of sPLA2 activity during early phase of reperfusion decreased the extent of injury and increased viability of cardiomyocytes (18, 21). Beside the secretoric *Pla2g2a* and *Pla2g5a*, myocardium express cytosolic PLA2 (cPLA2) which has been shown to be activated by CNH in normotensive rats (22). These isoforms cooperate in producing cardioprotective eikosanoid prostagladin E2 (23–25).

*Acer2* was more expressed after CNH in SHR-mt^BN^, which may suggest anti-apoptotic action associated with an improved energy balance and stronger infarct size-limiting effect shown previously (9). ACER2 is a Golgi complex associated alkaline ceramidase catalyzing the conversion of ceramide, with preferences to long- and very long-chain (VLC) unsaturated ceramides, to sphingosine, which in turn is phosphorylated to sfingosine-1-phosphate (S1P) (26, 27). S1P-activated signaling pathway leads to cell survival in opposite to ceramide which is associated with apoptosis (28).

### Cell Signaling

*Pkce* markedly declined only in progenitor SHR after CNH showing an interesting difference between strains which was unmasked by long-lasting hypoxic exposure. PKCε has been shown to play a role roles in myocardial ischemic tolerance and cardioprotection induced by CNH (29). Proposed mitochondrial targets of PKCε include components of electron transport chain (30) and mPTP pore where PKCε is supposed to form a complex with various proteins such as hexokinase2, adenine nucleotide translocase and voltage-dependent anion channel and inhibit the pathological function of the pore (31). Moreover, murine hearts with constitutively active PKCε were shown to preserve mitochondrial electron transfer coupling with maintained mitochondrial membrane potential and decreased cytochrome *c* release induced by reperfusion (32).

Similarly as P*kce* transcript, *Pparg* and *Pgc1* decline after CNH were also prevented by mitochondrial replacement in SHR-mt^BN^. These data suggest possible preservation of mitochondrial biogenesis (33) in SHR-mt^BN^ as PGC-1 may also indirectly control the expression of mitochondrial DNA (mtDNA) transcription via increased expression of mitochondrial transcription factor A (Tfam) 1 (NRF-1) (34, 35). PGC-1α may stimulate its own transcription by co-activation: PPARγ binding to the PGC-1α promoter (36). Activation of PPARγ promotes glucose uptake and triglyceride synthesis in adipose tissue and the resulting reduction in circulating glucose and FA may directly modulate cardiac PPARα and PPARδ activities (37, 38). PPARγ null mice showed a downregulation of manganese superoxide dismutase transcript and protein levels within the cardiomyocytes indicating an essential role of PPARγ in the antioxidant defense (39). Possible interplay between PPAR and PKC cannot be excluded (40, 41).

### Antioxidant Defense

Mitochondrial genome replacement also prevented the decreased gene expression of *Hmox2, Aco1* and *Prx6* induced by CNH in SHR and simultaneously increased *Cat* mRNA transcript. These changes may improve antioxidant defense of SHRmt^BN^ compared to SHR. Importantly, CNH increased inducible *Hmox1* transcript in both SHR strains. Accordingly, in non-hypertrophied left ventricles of normotensive rats, we observed CNH-induced increase of *Hmox1*, whereas upregulation of *Hmox2 and Aco1* was observed after cardioprotective regimen (8 h/day, 3 weeks) of intermittent normobaric hypoxia (12). Constitutively expressed HMOX2 possesses common antioxidant features (42) and *Hmox2* deletion led to phenotype characterized by increased pro-inflammatory and oxidative markers (43). Increased expression *of Cat* in SHR-mt^BN^ may contribute to H_2_O_2_ detoxifying in the myocardium within the peroxisomes. However, rat cardiac mitochondria were shown to contain CAT as well (44). CAT overexpressing transgenic mice are resistant to myocardial I/R injury (45).

PRX6 is primarily a member of cytosolic peroxiredoxins with two important functions (i) antioxidant defense and (ii) phospholipid homeostasis because of its PLA2 activity (46). Beside that PRX6 was shown to play an important role in the pathophysiology of type 2 diabetes (T2DM). The deficiency of *Prx6*^(−/−)^ impaired insulin signaling in mice, leading to reduction of muscle glycogen uptake (47). SHR-mt^BN^ have a lower skeletal muscle glycogen than SHR (8) in accordance with lower expression of *Prx6*. However, in the present study SHR responded to CNH by significant drop of *Prx6* transcript which was prevented in conplastic strain. These findings are in agreement with our previous data (8) demonstrating that selective replacement of the mitochondrial genome of the SHR with the mitochondrial genome of the BN rat influences several major metabolic risk factors of type 2 diabetes.

### Mitochondrial and Apoptosis Associated Genes

Mitochondrial biogenesis involves morphological changes in mitochondrial reticulum such as fusion and fission. Dynamin related protein coding by *Drp1* gene belongs to fission proteins, while *Mfn1* gene coding dynamin-related fusion protein mitofusin, which is involved in internal membrane fusion. Increased expression of both genes in SHR-mt^BN^ after CNH suggests more pronounced mitochondrial dynamics in this strain. Beside that, Drp1 has been shown to participate in peroxisomal fission process (48–50) which can also reflect the improved metabolic interplay between mitochondria and peroxisomes in SHR-mt^BN^ after CNH.

Traditionally, CASP3 is seen as the activator of apoptosis. Its activation starts the apoptotic cascade in pressure overload-induced heart failure (51). On the other hand, defective Akt activation in Casp3 KO mice was accompanied by impaired cell survival, increased apoptosis in stressed organs with marked lapse in their physiological functions (52). An increased level of Casp3 mRNA in the present study suggests an increase in the mitochondrial domain of the central apoptotic effector molecule in the form of precursor caspase 3 in SHR-mt^BN^ after CNH with the possibility of rapid response to stress stimuli (53).

In conclusion, the PCA analysis of Cq data clearly selected 14 important genes out of 48 ones that can be considered to contribute to the differences observed in myocardial ischemic tolerance between chronically hypoxic progenitor SHR and conplastic SHR-mt^BN^ strains. Most of them belong to glucose and lipid metabolism and were unmasked by adaptation to the cardioprotective regimen of CNH. The data also suggest an improvement in certain antioxidant, metabolic, and anti-inflammatory aspects induced by the replacement of SHR mitochondrial genome with that of normotensive BN rats.

## Author Contributions

IN performed experiments, analyzed data, interpreted data, and drafted and edited manuscript. DK performed experiments and edited manuscript. JN contributed to experimental design and the hypoxic model. MK performed experiments. MP and JŠ contributed the rat conplastic strain donor, and edited the manuscript. VK performed experiments and data analysis. FK contributed to the experimental model of hypoxia, data interpretation, and edited the manuscript. JZ contributed to experimental design, performed experiments, interpreted data, drafted and edited the manuscript.

### Conflict of Interest Statement

The authors declare that the research was conducted in the absence of any commercial or financial relationships that could be construed as a potential conflict of interest.
