# Supplementary material for: Cardioprotective Regimen of Adaptation to Chronic Hypoxia Diversely Alters Myocardial Gene Expression in SHR and SHR-mtBN Conplastic Rat Strains
Source: Front Endocrinol (Lausanne). 2019 Jan 22;9:809. doi: 10.3389/fendo.2018.00809 (PMC6350269; doi:10.3389/fendo.2018.00809)
Supplement: Supplementary file 1 [file Table_1.DOCX]

**Table 1:** Specific primers sequences of analyzed genes.

| Name of gene | Abbreviation of gene | Sequence (5' > 3') | | Area of the interest |
| --- | --- | --- | --- | --- |
| Hexokinase 1 | *Hk1* | F | *tct-ggg-ctt-cac-ctt-ctc-at* | Glucose metabolism |
|  |  | R | *atc-aag-att-cca-cag-tcc-agg-t* |  |
| Hexokinase 2 | *Hk2* | F | *cca-gca-gaa-cag-cct-aga-cc* |  |
|  |  | R | *aga-tgc-ctt-gaa-tcc-ctt-tg* |  |
| Pyruvate dehydrogenase kinase 3 | *Pdk3* | F | *cat-gta-ctc-aac-tgc-tcc-tcg-t* |  |
|  |  | R | *ttg-gca-agc-cat-aac-caa-at* |  |
| Pyruvate dehyrogenase phosphatase catalytic subunit 2 | *Pdp2* | F | *ccg-gtg-acg-tgc-agc-aat-ttt-atg-ac* |  |
|  |  | R | *ggc-tag-aat-gtt-gtc-tat-cca-gtc-acc* |  |
| Acyl-Coa dehydrogenase, long chain | *Acadl* | F | *gca-gtt-act-tgg-gaa-gag-caa* | Lipid metabolism |
|  |  | R | *ggc-atg-aca-ata-tct-gaa-tgg-a* |  |
| Alkaline ceramidase 2 | *Acer2* | F | *atg-tgt-gct-ttg-gcc-atg-t* |  |
|  |  | R | *cac-aca-cca-ctg-cct-tga-ac* |  |
| Fatty acid translocase | *Cd36* | F | *tga-gaa-gtc-tcg-aac-act-gag-g* |  |
|  |  | R | *tcc-aaa-cac-agc-ata-gat-gga-c* |  |
| Phospholipid phosphatase 1 | *Pap2a* | F | *agg-act-cac-ata-cga-ctc-tac-acg* |  |
|  |  | R | *tca-tga-ttc-ctt-gcg-tag-ctc* |  |
| Phospholipase A2 group IIA | *Pla2g2a* | F | *gtg-acc-tac-aag-ttc-tcc-tac-cg* |  |
|  |  | R | *tta-tcg-cac-tgg-cac-agc* |  |
| Phospholipase A2 group IVA | *Pla2g4a* | F | *tct-ggc-tca-cag-aat-aaa-ggt-tc* |  |
|  |  | R | *ctc-aca-atg-tgc-ttt-gct-gta-a* |  |
| Phospholipase A2 group V | *Pla2g5* | F | *tac-cga-ggc-tgc-cca-tag* |  |
|  |  | R | *cgc-ttc-att-tct-tgg-gtt-ct* |  |
| Sphingomyelin synthase 2 | *Sgms2* | F | *act-tac-aca-agc-ccc-act-gaa* |  |
|  |  | R | *ggg-ttt-acc-ctt-gcc-att-tt* |  |
| Sphingomyelin phosphodiesterase 2 | *Smpd2* | F | *ccc-atc-tgc-acg-ctg-agt-a* |  |
|  |  | R | *tgc-ttt-ctt-gga-tgt-gtg-gt* |  |
| Serine-palmitoyl-coa transferase 1 | *Sptlc1* | F | *ctc-agg-cac-gct-act-tgg-ac* |  |
|  |  | R | *ggt-gac-cac-aac-cct-gat-g* |  |
| Aconitase 1 | *Aco1* | F | *ttg-ctg-tgt-ctg-aga-ttg-aaa-ag* | Antioxidant defense |
|  |  | R | *ctt-gaa-aac-ctt-taa-atc-ctt-gct* |  |
| Aconitase 2 | *Aco2* | F | *cgc-ctt-aca-gcc-tac-tgg-tc* |  |
|  |  | R | *ggc-aga-ggc-cac-atg-gta* |  |
| Catalase | *Cat* | F | *cag-cga-cca-gat-gaa-gca* |  |
|  |  | R | *ggt-cag-gac-atc-ggg-ttt-c* |  |
| Glutathione-disulfide reductase | *Gsr* | F | *ttc-ctc-atg-aga-acc-aga-tcc* |  |
|  |  | R | *tga-aag-aac-cca-tca-ctg-gtt-a* |  |
| Hemoxygenase 1 | *Hmox1* | F | *gtc-aag-cac-agg-gtg-aca-ga* |  |
|  |  | R | *ctg-cag-ctc-ctc-aaa-cag-c* |  |
| Hemoxygenase 2 | *Hmox2* | F | *tac-ggc-acc-aga-aaa-gga-aa* |  |
|  |  | R | *gtg-ctt-cct-tgg-tcc-ctt-c* |  |
| Monoaminooxidase A | *Maoa* | F | *tgg-tat-cat-gac-cca-gta-tgg-a* |  |
|  |  | R | *tgt-gcc-tgc-aaa-gta-aat-cct* |  |
| Peroxiredoxin 6 | *Prx6* | F | *ttg-att-gct-ctt-tca-ata-gac-tct-g* |  |
|  |  | R | *ctg-cac-cat-tgt-aag-cat-tga* |  |
| Thioredoxin reductase 1 | *Txrd1* | F | *agc-acc-att-ggc-atc-cac* |  |
|  |  | R | *cca-cac-tgg-ggc-tta-acc-t* |  |
| Thioredoxin reductase 2 | *Txrd2* | F | *gca-cat-ggt-gaa-gct-acc-tag-a* |  |
|  |  | R | *gct-cca-tcc-aca-tct-tct-cag* |  |
| BCL2 associated X, apoptosis regulator | *Bax* | F | *gtg-agc-ggc-tgc-ttg-tct* | Genes associated with apoptosis |
|  |  | R | *gtg-ggg-gtc-ccg-aag-tag* |  |
| BCL2 apoptosis regulator | *Bcl2* | F | *gta-cct-gaa-ccg-gca-tct-g* |  |
|  |  | R | *ggg-gcc-ata-tag-ttc-cac-aa* |  |
| Caspase 3 | *Casp3* | F | *ccg-act-tcc-tgt-atg-ctt-act-cta* |  |
|  |  | R | *acc-tgg-ttc-atc-atc-act-aat-cac* |  |
| X-Linked inhibitor of apoptosis | *Xiap* | F | *gct-tgc-aag-agc-tgg-att-tt* |  |
|  |  | R | *tgg-ctt-cca-atc-cgt-gag* |  |
| CRLS, beclin 1 | *Atg6* | F | *cag-gcg-aaa-cca-gga-gag* | Genes associated with mitochondria |
|  |  | R | *cga-gtt-tca-ata-aat-ggc-tcc-t* |  |
| Citrate synthase | *Cs* | F | *aga-cat-cgg-ttc-ttg-atc-ctg* |  |
|  |  | R | *gcc-aaa-ata-agc-cct-cag-gt* |  |
| Dynamin-related factor | *Drp* | F | *gct-ggt-cca-cgt-ttc-acc* |  |
|  |  | R | *ccc-cat-tct-tct-gct-tca-ac* |  |
| Mitofussin 1 | *Mfn1* | F | ***caa-act-gca-gcc-acc-aag-t*** |  |
|  |  | R | ***gtt-ggc-aca-gtc-gag-caa*** |  |
| OPA1, mitochondrial dynamin like gtpase | *Opa1* | F | ***gga-ttt-ctt-cac-tgc-ggg-ta*** |  |
|  |  | R | ***cgg-atc-cat-gat-ctg-ttg-c*** |  |
| PPARG coactivator 1 alpha | *Pgc1a* | F | *aaa-ggg-cca-agc-aga-gag-a* |  |
|  |  | R | *gta-aat-cac-acg-gcg-ctc-tt* |  |
| Adenylate cyclase 5 | *Adcy5* | F | *ggg-aga-acc-agc-aac-agg* | Cell signalling |
|  |  | R | *cat-ctc-cat-ggc-aac-atg-ac* |  |
| Adenylate cyclase 6 | *Adcy6* | F | *atg-aga-tca-tcg-cgg-act-tt* |  |
|  |  | R | *gcc-atg-taa-gtg-cta-ccg-atg* |  |
| Adrenoceptor beta 1 | *Adrb1* | F | *aga-gca-gaa-ggc-gct-caa-g* |  |
|  |  | R | *agc-cag-cag-agc-gtg-aac* |  |
| MDM2 proto-oncogene | *Mdm2* | F | *cag-aaa-ctt-agt-ggt-tgt-aag-tca-aca* |  |
|  |  | R | *ttc-agg-tca-ctc-cca-cct-tc* |  |
| Protein kinase C delta | *Pkcd* | F | *caa-gaa-gaa-caa-cgg-caa-gg* |  |
|  |  | R | *tgc-aca-cac-atc-agcacct* |  |
| Protein kinase C epsilon | *Pkce* | F | *aaa-cac-cct-tat-cta-acc-caa-ctc-t* |  |
|  |  | R | *cat-att-cca-tga-cga-aga-aga-gc* |  |
| Peroxisome proliferator activated receptor alpha | *Ppara* | F | *tgc-gga-cta-cca-gta-ctt-agg-g* |  |
|  |  | R | *gct-gga-gag-agg-gtg-tct-gt* |  |
| Peroxisome proliferator activated receptor gamma | *Pparg* | F | *ccc-aat-ggt-tgc-tga-tta-ca* |  |
|  |  | R | *gga-cgc-agg-ctc-tac-ttt-ga* |  |
| PKR, tumor protein p53 | *P53* | F | *aga-gag-cac-tgc-cca-cca* |  |
|  |  | R | *aac-atc-tcg-aag-cgc-tca-c* |  |
